# Patients with Controlled and Uncontrolled Type 2 Diabetes: Systemic Oxidative Stress and Systemic Inflammation as Predictors of Peripheral and Central Neuropathy

**DOI:** 10.1002/alz.084887

**Published:** 2025-01-09

**Authors:** Thura Tun Oo, Max Skarr, Yee Yee Mon, Kyi Kyi Htwe

**Affiliations:** ^1^ Department of Biomedical Sciences, College of Medicine Rockford, University of Illinois, Rockford, IL USA; ^2^ Clinical Research Center, Department of Medical Research, Yangon Myanmar

## Abstract

**Background:**

Diabetes mellitus increases the risk of cognitive decline and neuronal degeneration. In diabetes, persistently elevated blood sugar levels cause not only the generation of reactive oxygen species (ROS), but also systemic inflammation (1). This raises an intriguing question: do patients with controlled or uncontrolled diabetes exhibit similar levels of oxidative stress and systemic inflammation as reliable predictors of peripheral neuropathy and cognitive decline?

**Method:**

In 2019, 150 participants with diabetes mellitus who had been diagnosed for more than 5 years were voluntarily enrolled. Blood was obtained to measure systemic oxidative stress and inflammation. Vibrometer and Mini‐Mental State Examination (MMSE) were used to assess peripheral and central neuropathy, respectively. The association with peripheral and central neuropathy was evaluated using multivariate logistic regression analyses.

**Result:**

Using HbA1C (6.5%) targets for categorization, 60% of participants were controlled diabetes patients and 40% were uncontrolled patients. The plasma malondialdehyde level was significantly higher in patients with uncontrolled diabetes than patients with controlled diabetes (63% Vs. 23%, P < 0.05). The C‐reactive protein (CRP) level significantly higher in patients with uncontrolled diabetes than patients with controlled diabetes (70% Vs. 35%, P < 0.05). In patients with uncontrolled diabetes, there was a correlation between the level of plasma malondialdehyde and sensory neuropathy as well as cognitive decline (r = 0.774, P < 0.05 and r = 0.734, P < 0.05, respectively), whereas we found the correlation between the level of CRP and sensory neuropathy as well as cognitive decline (r = 0.873, P < 0.05 and r = 0.688, P < 0.05, respectively). Both sensory neuropathy and cognitive decline were uncorrelated with the level of plasma malondialdehyde in patients with controlled diabetes, and neither was there a correlation with the level of CRP.

**Conclusion:**

This study demonstrated that systemic oxidative stress and systemic inflammation are reliable clinical predictors of peripheral and central neuropathy in uncontrolled diabetes, but not in controlled diabetes.

